# The role of interleukin-1β in type 2 diabetes mellitus: A systematic review and meta-analysis

**DOI:** 10.3389/fendo.2022.901616

**Published:** 2022-07-27

**Authors:** Hend Alfadul, Shaun Sabico, Nasser M. Al-Daghri

**Affiliations:** ^1^Chair for Biomarkers of Chronic Diseases, Biochemistry Department, College of Science, King Saud University, Riyadh, Saudi Arabia; ^2^Biochemistry Department, College of Science, King Saud University, Riyadh, Saudi Arabia

**Keywords:** type 2 diabetes mellitus, interleukin 1β, inflammasome, inflammation, inflammatory disease, cytokines

## Abstract

Type 2 diabetes mellitus (T2DM) is a multifactorial non-communicable disease that is characterized by insulin resistance and chronic sub-clinical inflammation. Among the emerging inflammatory markers observed to be associated with β-cell damage is interleukin 1β (IL1β), a proinflammatory cytokine that modulates important metabolic processes including insulin secretion and β-cell apoptosis. The present systematic review and meta-analysis gathers available evidence on the emerging role of IL1β in T2DM. PubMed and Embase were searched for human studies that assessed 1L1β in T2DM individuals from 2016-2021. Thirteen studies (N=2680; T2DM=1182, controls=1498) out of 523 were included in the systematic review and only 3 studies in the meta-analysis. Assays were the most commonly used quantification method and lipopolysaccharides as the most common stimulator for IL1β upregulation. Random and fixed effects meta-analysis showed non-significant mean differences of IL1β concentrations between the T2DM and controls. Given the high heterogeneity and small subset of studies included, caution is advised in the interpretation of results. The present systematic review and meta-analysis highlights the limited evidence available that could implicate 1L1β as a potent biomarker for T2DM. Standardization of 1L1β assays with larger sample sizes are encouraged in future observational and prospective studies.

## Introduction

According to the International Diabetes Federation (IDF), 451 (CI 367.5–585.5) million people aged 18–99 years lived with diabetes as of 2017 (1). This figure is predicted to rise to 693 (CI 521.9–902.5) million by 2045, or 281% higher from data taken in 2000 (1). Type 2 diabetes mellitus (T2DM) reduces life expectancy by as much as one decade (2). A particular feature of T2DM is chronic hyperglycemia caused by genetic and environmental factors with concomitant defects in both β-cells’ insulin action and secretion (in fat, muscle, liver and elsewhere) (3). T2DM is considered a chronic inflammatory disease with high circulating levels of tumor necrosis factor (TNF), interleukins and adipokines (4).

Important sensors of T2DM metabolic dysfunction and β-cell dysregulation are known as inflammasomes. Inflammasomes are cytosolic macromolecular complexes that contain a sensing element that can activate an inflammatory response to an array of signals (5). The most extensively investigated inflammasome complex is the NLRP3 inflammasome (5). It is made up of the sensing protein (NLRP3), the adaptor protein; apoptosis-associated speck-like protein containing a caspase recruitment domain (ASC), and the effector protein; pro-caspase-1, forming an intracellular multi-protein complex that activates the synthesis of Interleukin-1β (IL-1β) and Interleukin-18 (IL-18) (6). It also represents an important link between the immune system and the metabolic system (7).

In general, it may be theorized that inflammasomes have an important role in the activation of chronic inflammation as observed in pro-inflammatory related diseases. Thus, the association of inflammasomes in the progress of pro-inflammatory related diseases is reasonable (4). Particularly, over expression of NLRP3 inflammasome is now identified as a key player in the development of numerous inflammatory and autoimmune diseases which include atherosclerosis, neuro-degenerative diseases, obesity and T2DM (8–11). T2DM is caused partly by a state of low-grade inflammation due to excess nutrients and excess metabolic stress (metaflammation). Evidence also indicates a direct key function of the innate system in the destruction of β-cells (12) and impaired insulin secretion (13). Accumulating evidence points out that the inflammation status of T2DM islets has two key characteristics: elevated cytokine levels and increased activation of immune cells, mostly macrophages (14).

**Figure 1:**
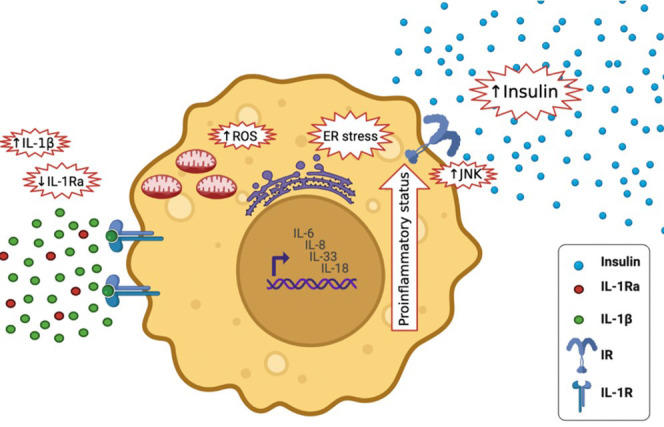
IL-1β Synthesis and Regulation. NLRP3 inflammasome is activated *via* two signals; the priming signal which activates the transcription factor NF-κB leading to the upregulation of NLRP3, pro-IL-1β and IL-1Ra mRNA and the activation signal which stimulates NLRP3 inflammasome complex assembly and activation, assembled caspase-1 undergoes self-cleavage and activation. Active caspase-1 triggers the activation and release of IL-1β. IL-1β regulates its own synthesis in an autoregulatory feed-back loop (15).

IL-1β, a pro-inflammatory cytokine, has been underlined as a strong driver of β-cell damage. β-cell macrophages are the main contributors to IL-1β production. IL-1β levels, being a potent pro-inflammatory driving cytokine, is kept under strict regulation by IL-1 receptor antagonist (IL-1Ra). During inflammation, macrophages are the main source of IL-1β/IL-1Ra, making both cytokines in an autoregulatory feed-back loop (**Figure 1**) (15). In comparison to other cells, β cells express an abundant number of interleukin 1 receptor (IL-1R). Thus, the balance in IL-1β/IL-1Ra levels is crucial in defining the response of β-cells and ultimately the progression of T2DM (15). IL-1β encompasses various functions in regulating inflammatory responses and metabolism; it can regulate insulin secretion and promote β cell apoptosis which can eventually lead to T2DM (4, 16). Chronically elevated IL-1β levels in obese and T2DM individuals cause β-cell dysfunction (16). IL-1β signaling events induce an acute phase response, low blood pressure, dilation of the blood vessels, and fever, which can ultimately result in large inflammatory events (17). Still, the role of inflammation, immune cells and interleukins in particular, in the pathogenesis of T2DM, remains a gray area in the field that requires further evaluation (18).

In the current systematic review and meta-analysis, we aimed to determine whether there is an association between T2DM and circulating IL-1β based on available evidence from 2016-2021.

## Methods

The current systematic review followed the PRISMA (Preferred Reporting Items for Systematic Review and Meta-Analyses) guidelines (19). **Figures 1** and **6** were created using BioRender software (BioRender, Toronto, ON, Canada).

### Literature search

A systematic search in PubMed and Embase was done last 5 September 2021 using the keywords [Interleukin-1 beta AND type 2 diabetes mellitus].

### Study selection

The inclusion criteria were as follows:

1. Case-control, observational studies and clinical trials conducted in human subjects that assessed IL-1β in participants.

2. Studies written in the English language.

3. Studies with available free full text source.

4. Studies with adult participants (19+ years old).

5. Studies conducted in the last 5 years (2016-2021).

#### The exclusion criteria were as follows

1. Review articles.

2. Studies among nonhuman subjects.

3. Pre-existing disease in participants other than T2DM.

4. Studies that didn’t measure IL-1β in participants.

5. Sample type: stem cells, saliva, human umbilical cord MSCs, purified human islets co-cultured, animal and rat cell line

6. Sites that couldn’t be accessed.

7. Studies that did not discriminate or exclude patients based on medical and medication history.

8. Studies that did not include T2DM patients.

### Data extraction

Titles and abstracts of all articles were screened for eligibility. Full articles were retrieved for eligible abstracts if available and evaluated. For the evaluation of studies, CASP tool was used to evaluate the quality of all included studies (**Supplementary 1**). For the meta-analysis, review manager 5.4 https://training.cochrane.org/online-learning/core-software-cochrane-reviews/revman/revman-5-download was used. Only case control studies were included. Two independent investigators (HA and SS) extracted pertinent data from eligible studies using a standard MS Excel spreadsheet with the senior investigator (NA) supervising entry. Information extracted included the full citation of the article, country where the investigation was done, study design, sample size, age, body mass index (BMI), number of males and females if available, methods used to assess IL-1β and outcome.

## Results

**Figure 2:**
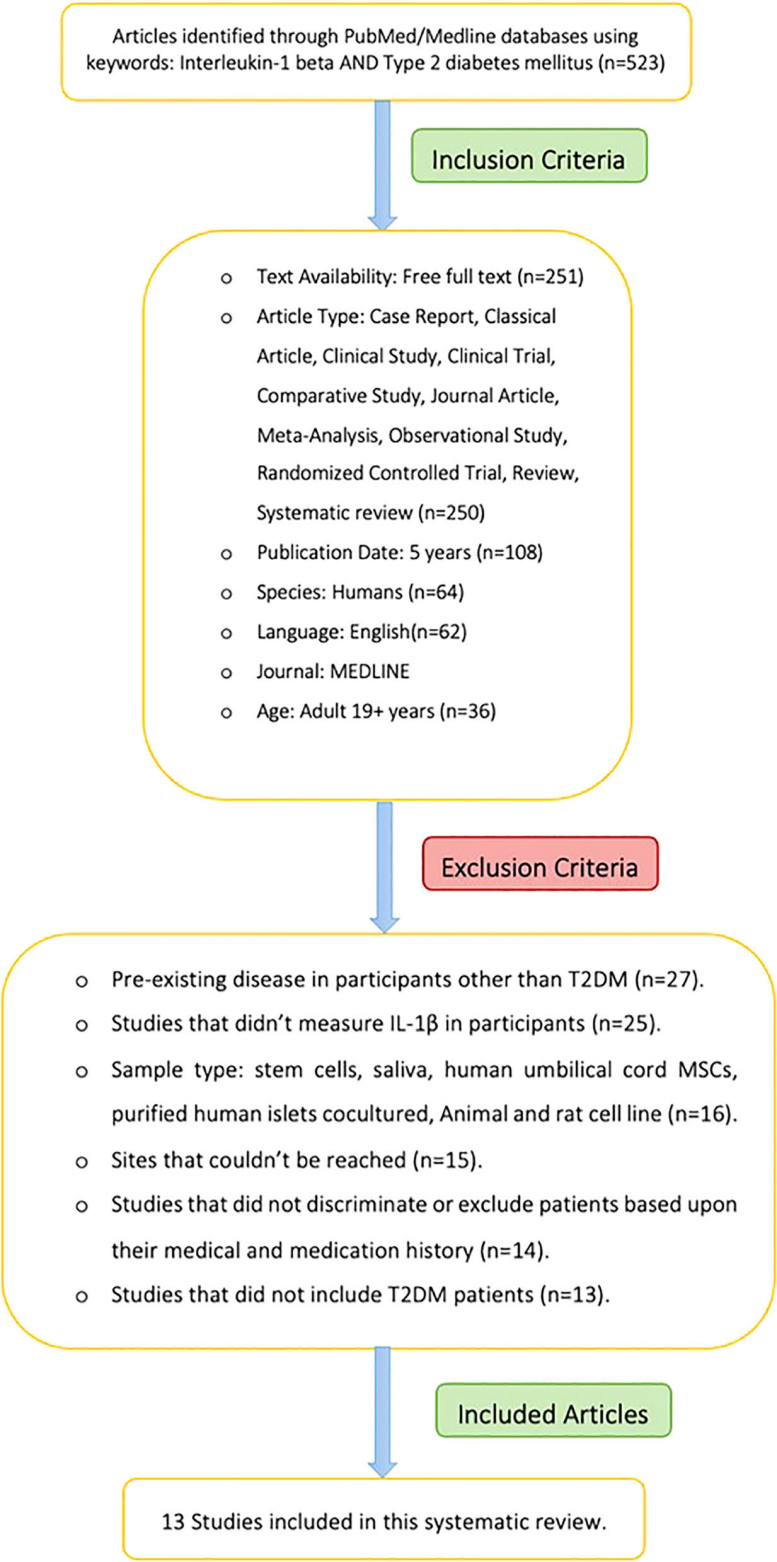
Workflow of systematic review.

The systematic search strategy returned 523 articles. After applying the selection criteria, 13 articles were included (**Figure 2**). These studies included a total of 2680 adults, 1182 were T2DM cases (mean age 54.2 ± 6.7 years) and 1498 were controls (mean age 50.2 ± 6.2 years). Sample sizes ranged from 22 (20) to 1643 individuals (21). Mean BMI in the T2DM group (N=10 studies) was 26.9 ± 3.2kg/m^2^, while the mean BMI in controls (N=8 studies) was 24.3 ± 1.3kg/m^2^.

A total of 13 eligible studies (10 case control studies, 1 Prospective Randomized Controlled study, 1 interventional study and 1 prospective controlled study) were included in this systematic review and meta-analysis. Four studies were conducted in Europe, six in Asia and three in South America (**Table 1**). The mean and standard deviation of the T2DM group and the HC group was only mentioned in three studies.

**Table 1:** Summary of included studies.

	Study Setting	Population (M/F)	Samples/Kit Used	Outcome	Strengths and Limitations
1	(15) Armenia	T2DM: N= 35 (15/20) Age= 53.8 (46-69) BMI= 25.7 ± 3.5 HC: N= 31(16/15) Age= 51.9 (40-67) BMI= 23.5 ± 2.6	Plasma samples and supernatants of cultured PBMCs/ELISA MAX Deluxe kit (Biolegend, USA)	* IL-1β plasma levels are increased in T2DM patients (P = 0.038). * LPS stimulation increased release of IL-1β compared to unstimulated samples except female patients. LPS induced IL-1β production by PBMCs from HC and T2DM males (*P* = 0.01 and *P* = 0.03, respectively) and in HC females (*P*< 0.001).	* Hypomethylation of IL1RN and NFKB1 promotor regions in PBMCs of T2DM group and not in HC group.
2	(22) Armenia	T2DM: N= 17 (7/10) Age= 45.4 ± 10.1 BMI= 27.3 ± 2.4 HC: N= 15 Age= NM BMI= NM	Neutrophils Protein: ELISA MAX Deluxe kits (Biolegend, UK) mRNA: Hs01555410_m1 (AppliedBiosystems, Foster City CA, USA)	* Freshly isolated neutrophils from T2DM group did not exhibit significant differences in mRNA expression of IL-1β gene as compared to HC group. * sFasL significantly increased mRNA levels of IL-1β in neutrophils from T2DM group. * Protein levels of IL-1β were not affected by the sFasL treatment (P>0.05).	* sFasL exhibited proinflammatory effect and induced mRNA levels of caspase-1, NF-κB and IL-1β. * Did not assess the impact of different therapy (gliclazide/metformin) on activation status of neutrophils. * HC group were not perfectly age-matched with the T2DM group. * Small sample size.
3	(16) Vietnam	T2DM: N= 73(40/33) Age= 55.7 ± 11.5 BMI= NM HC: N= 57 (12/45) Age= 48.5 ± 6.8 BMI= NM	Serum/AviBion Human IL-1β (Orgenium, Helsink,Finland)	* IL-1β levels were significantly increased in T2DM group compared with the HC group. (P < 0.0001). * IL‐1β levels were not significantly different between the overweight and non‐overweight T2DM group. * Adiponectin levels were negatively correlated with IL‐1β levels.	* Levels of adiponectin and IL-1β are significantly modulated during the development of overweight and T2DM * Adiponectin levels are correlated with several pro‐inflammatory cytokines including IL-1β and with clinical parameters of obesity and T2DM. * Did not mention if age and sex were matched in the T2DM and the HC group.
4	(23) China	T2DM: N= 70 (45/25) Age= 51.31 ± 9.25 BMI= 24.84 ± 2.74 HC: N= 32 (13/19) Age= 52.41 ± 8.57 BMI= 23.71 ± 2.60	Blood/enzyme immunoassay kits (R&D systems, Minneapolis, MN)	* No significant difference in IL-1β protein levels between the T2DM and the HC group. * No significant difference in IL-1β levels between the acarbose-treated and metformin-treated T2DM subgroups. * No significant differences in IL-1β levels were observed between the T2DM patients and the HC after 12 months of treatment. * After 6 months or 12 months of treatment, the variation margins of IL-2, IL-6, IL-1β, TNF-α and ferritin levels were similar between the acarbose-treated and metformin-treated T2DM subgroups.	* Chronic inflammatory status is not improved as quickly as glucose metabolism and required a long-lasting therapy (>12month). * Small sample size. * No placebo/control group. * Did not determine whether the effect of a longer term (>12month) treatment on the levels of inflammatory factors reduced vascular complications.
5	(24) India	T2DM: N= 80 Age= NM BMI=NM HC: N= 80 Age= NM BMI= NM	Serum/Cytometric Bead Array (CBA) method 558279 (IL-1β flex set)	* IL-1β levels were significantly increased in the T2DM group (P= 0.019) in early middle aged (31-40 years) and not effected in late middle aged (41-50 years) (P=0.167). * IL-1β did not show a significant association with ageing among the T2DM group.	* Oxidative stress and proinflammatory markers (including IL-1β) were significantly increased in the early-middle-aged (31-40 years) T2DM group compared to the HC group. * One-point single-centered study.
6	(21) India	T2DM: N= 558 (293/265) Age= 57.29 ± 9.41 BMI= 26.18 ± 5.38 HC: N= 1085 (553/532) Age= 37.72 ± 17.19 BMI= 23.74 ± 6.04	Whole blood/quantitative PCR using SYBR Green method and their respective forward and reverse primers (Eurofins, Bangalore, India) Real-time PCR (LightCycler480 Real- Time PCR, Roche) was performed in duplicate in 10 μl volume using LightCycler480 SYBR Green I Master mix (Roche Diagnostics GmbH, Mannheim, Germany) as per the instruction manual	* No difference in the genotype and allele frequencies (IL-1β -511C/T polymorphism) was observed between the T2DM and the HC group. * There was also no difference between genotype frequency TT vs TC while TT vs CC frequency differed significantly in the T2DM and the HC group. * mRNA levels of IL-1β were significantly higher in the T2DM group compared to the HC group. * 4-fold higher expression of IL-1β transcript in the T2DM group compared to the HC group.	* The study proposes the possible involvement of IL-1β polymorphisms for genetic susceptibility to T2DM in Gujarat population. * Limited to Gujarat population.
7	(25) Korea	T2DM: N= 141(60/81) Age= 56.5 ± 10.7 BMI= 23.5 ± 3.5 HC: N= 22 (11/11) Age= 51.6 ± 6.0 BMI= 23.0 ± 2.6	Plasma/DLB50, R&D systems	* IL-1β levels were significantly increased in patients with T2DM (P <0.001). * The circulating cell-free mitochondrial DNA (ccf-mtDNA) levels were increased in patients with type 2 diabetes. * Weak correlation between the elevated ccf-mtDNA levels and IL-1β levels in plasma from patients with type 2 diabetes.	* Provides evidence that elevated ccf-mtDNA levels may contribute to chronic inflammation in patients with type 2 diabetes. * Small sample size of HC group.
8	(26) China	T2DM: N= 56(28/28) Age= 50.95 ± 12.48 BMI= 25.60 ± 3.69 IGT: N= 35 (17/18) Age= 51.85 ± 8.57 BMI= 23.88 ± 3.20 HC: N= 45 (22/23) Age= 52.29 ± 12.16 BMI= 23.16 ± 1.98	Serum/Human ELISA kit; USCN, Wuhan, China	* IL-1β levels were significantly increased in the IGT and T2DM groups compared to the HC group (P <0.001). * IL-1β levels were significantly increased in the T2DM group compared to the IGT group (P <0.001). * Serum adipsin levels was negatively correlated with IL‐1β levels.	* This is the first study to investigate the relationship between serum adipsin levels and the first phase of insulin secretion in humans with different glucose tolerance. * Small sample size.
9	(27) Italy	T2DM: N= 38 Age= 61.2 ± 6.8 BMI= NM HC: N= 31 Age= 61.9 ± 11.3 BMI= NM	Blood monocyte/ELISA BioSource, Nivelles, Belgium	* No significant differences in the concentration of IL-1β released by monocytes cultured in (low, normal, and high glucose concentrations, with and without LPS stimulation) between the T2DM and the HC group. * Low glucose concentration increased IL-1β production by monocytes from the T2DM and HC group pre and post LPS stimulation. * IL-1β production was unaffected by high glucose concentrations in both the T2DM and the HC group.	* First study to provide evidence that *low glucose* concentrations may be a powerful stimulus for proinflammatory cytokine expression (IL-1β) by human monocytes. * Small sample size.
10	(28) Spain	T2DM: N= 31 (31/0) Age= 48 (45,58) BMI= 33.3 (27.5, 46.7) HC: N= (27/0) Age= 50 (40,53) BMI= 24.8 (23, 26.6)	Venous blood, PBMC mRNA: Real-time quantitative PCR technology Protein: Immun-StarTM Western CTM Chemi- luminescence Kit (cat# 170–5070; Bio-Rad	* I-1β mRNA and protein levels were significantly increased in the T2DM group compared to the HC group. * This induction was notably suppressed after 24 h treatment with recombinant β 1-, β 3- and β 6-conglutin proteins.	* 1, β3, and β6 proteins can regulate the level of mRNA and protein synthesis of crucial genes involved in the insulin molecular signaling pathway, thereby modulating the activation and response of these regulatory genes leading to variation in insulin-mediated plasma glucose levels. * Small sample size. * All participants were male.
11	(20) Brazil	T2DM: N= 10 (0/10) Age= 70 ± 3 BMI= 28.4 ± 1.4 HC: N= 12 (0/12) Age= 62.9 ± 1.8 BMI= 26.6 ± 1.2	Plasma and neutrophils/DuoSet ELISA kits (R&D Systems, Minneapolis, MN, USA)	* Four months of dance training did not modify plasma IL-1β levels in the T2DM and the HC group. * Before training there was no difference for neutrophil IL-1β production in the T2DM and the HC group. * After training there was no difference in neutrophil IL-1β production between the T2DM and the HC group following LPS stimulation.	* This research was the first to investigate neutrophil function and death after a moderate-intensity dance program in people with T2DM. * These findings may represent a useful tool to design nonpharmacological strategies to reduce inflammation and improve neutrophil clearance in patients with T2DM. * Small sample size. * All participants were female.
12	(29) Mexico	T2DM: N= 43 (15/28) Age= 53 [30-68] BMI= 26 [23-47] HC: N= 26 (9/17) Age= 48 [33-65] BMI= 26 [23-33]	Whole blood monocytes/ Cytometric bead array (BD Bioscience)	* No significant difference in IL-1β production in response to Pam3Cys, LPS and M. tuberculosis infection between the T2DM and the HC group. * Decreased IL-1β production in monocytes cultured in high glucose for 24 h and after stimulation with LPS.	* Provides evidence that the presence of high glucose concentrations decreased cytokine production and increased the intracellular growth of M. tuberculosis in monocytes. * Small sample size. * No mention if age and sex were matched in the T2DM and the HC group.
13	(30) Argentina	T2DM: N= 30(23/7) Age= 48.17 (23–69) BMI= 33.17 (30.82–38.64)	Serum and mononuclear leucocyte mRNA levels/ chemiluminescence (Immulite – Siemens, DPC) Quantitative real time PCR with the StepOne system (Applied Biosystems)	* At baseline, serum IL-1β levels positively correlated with FPG. * Significant association between T allele (CT and TT) and lower IL-1β mRNA expression. * IL-1β mRNA and protein levels had no differences with pre-intervention time. * Sharper decreases in FPG and HbA1c after treatment were associated with greater reductions in IL-1β protein levels.	* First follow-up study evaluating IL-1β mRNA expression and serum levels in a hyperglycemic T2DM group and after glycemic normalization treatment. * Short post-intervention study time. * This study was carried out in a population with different proportions of men and women.

T2DM, Type 2 Diabetes Mellitus; N, Number; BMI, Body Mass Index; PBMC, Peripheral Blood Mononuclear Cell; ELISA, Enzyme Linked Immunosorbent Assay; IL-1β, Interleukin 1 Beta; LPS, Lipopolysaccharides; IL1RN, Interleukin 1 Receptor Antagonist Gene; NFKB1, Nuclear Factor Kappa B Subunit 1 Gene; mRNA, Messenger RNA; sFasL, Soluble Fas Ligand; HC, Healthy Control; PCR, Polymerase Chain Reaction; SYBR, Synergy Brands; ccf-mtDNA, Circulating Cell Free Mitochondrial DNA; IGT, Impaired Glucose Tolerance; NGT, Normal Glucose Tolerance; NM, Not Mentioned; Pam3Cys, Palmitylated N-acyl-S-diacylglyceryl Cysteine; M. Tuberculosis Mycobacterium Tuberculosis; FPG, Fasting Plasma Glucose.

### Meta-analysis

**Figure 3:**
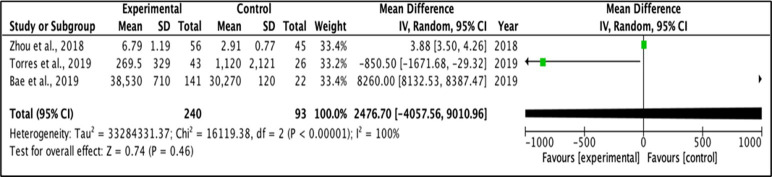
Forest plot for analysis of IL-1β levels.

**Figure 4:**
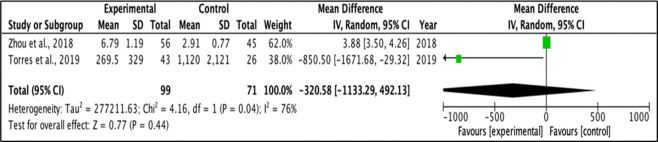
Forest plot for analysis of IL-1β levels.

A random effect model was applied for the mean difference of change between two groups. Meta-analysis was performed for outcomes examined in at least two studies, with provided mean values and standard deviation for each group. I-square test was used to measure heterogeneity between studies, and a value > 30·0 was considered to reflect high heterogeneity. **Figures 3** and **Figure 4** represent forest plots showing individual and pooled mean differences (95% CI) of IL1β concentration in T2DM and control subjects. The forest plots provided non-significant mean differences of IL1β concentration between the T2DM and controls with the pooled mean difference of 2476.7 (-4057.56 – 9010.96) and -320.58 (-1133.29 – 492.13) after removing the study of Bae and colleagues (2019) due to extremely high heterogeneity, as shown in **Figures 3** and **Figure 4** respectively. Both I^2^ statistics which were well above the threshold of 50% indicate heterogeneity among the included studies.

#### IL-1β protein

IL-1β protein levels were assessed in 12 studies, with no uniformity in assays used for assessment (**Table 1**). Two studies observed significantly higher IL-1β protein levels in the T2DM group than controls (15, 25). Protein levels of IL-1β were unaffected by the sFasL treatment in both T2DM and control groups (22). In the study of Tong and colleagues (2017), IL‐1β protein levels didn’t differ between the overweight and non‐overweight T2DM groups. However, in the control groups, IL‐1β levels were higher in the overweight group than the non‐overweight group (16). In one trial, 6 months of acarbose or metformin treatment, IL-1β protein levels were substantially lower in the T2DM group (23). With regards to age, Banerjee et al. (24) recorded significantly higher IL‐1β protein levels in the younger (31-40 years) T2DM group versus age-matched controls and no difference in G2 (41-50 years) T2DM group versus G2 controls. Zhou et al. (26) recorded elevated IL‐1β protein levels in both the impaired glucose tolerance (IGT) and T2DM groups than controls. Piarulli et al. (27) recorded elevated IL‐1β protein levels in monocytes cultured with low-glucose levels with and without LPS stimulation, which was not the same for normal/high glucose culture. Lima-Cabello et al. (28) recorded elevated IL‐1β protein levels in the T2DM group which decreased after day 1 of incubation with β1, β3, and β6 purified β-conglutin proteins. Borges et al. (20) observed no substantial difference in neutrophil IL‐1β levels in the T2DM group and controls pre/post-dance training intervention. Torres et al. (29) also observed no substantial difference in monocyte IL‐1β protein levels in T2DM and controls post Pam3Cys or LPS, or M. tuberculosis infection activation. Circulating IL‐1β decreased after high-glucose culture for day 1 and post activation with Pam3Cys or LPS or M. tuberculosis infection for 1 day. Iglesias Molli et al. (30) no substantial difference in IL‐1β protein levels pre/post pharmacological treatment and changes in regimen was observed. However, the higher reduction in fasting plasma glucose (FPG) and hemoglobin A1c (HbA1c) after treatment were associated with sharper reduction in IL-1β protein levels.

#### IL-1β mRNA

IL-1β mRNA levels were assessed in 4 studies. Two studies did not observe a substantial difference in mRNA levels between T2DM and controls. (21) observed that IL-1β mRNA levels were 4 times higher in the T2DM group than controls. sFasL stimulation significantly increased mRNA levels of IL-1β in neutrophils in the T2DM group. Elevated IL-1β mRNA levels were notably suppressed after day 1 of incubation with β1, β3, and β6 purified β-conglutin proteins (28).

### Intervention

In the study of Margaryan et al. (15), participants were receiving nutritional therapy recommendations and T2DM patients were receiving gliclazide and metformin. Furthermore, in an earlier investigation of Margaryan et al. (22), all T2DM participants were on diet therapy and oral hypoglycemic agents (N=5 gliclazide and N=12 metformin). In Tong et al. (16), participants newly diagnosed T2DM participants were either on gliclazide MR and/or a low dose of insulin injection. In Mo et al. (23), participants received four weeks of lifestyle therapy based on the Chinese diabetes management guidelines, with T2DM participants randomly assigned (1:1) to receive metformin or acarbose treatment. In Banerjee et al., (2020), most participants were on metformin in combination with other class of hypoglycemic drugs. In Zhou et al. (26), all the participants were placed on a diet consisting of 150 g of carbohydrate/day for 3 days before the test. In Piarulli et al. (27), 10 T2DM patients were on statins, 26 on antihypertensive drugs, and 9 on aspirin 100 mg/day. In Borges et al. (20), participants received dance training classes that consisted of 60 min of exercise carried out twice a week for 4 months. Four participants from the control group were on lipid-lowering agents, 1 was taking ACE inhibitor, 3 on beta-blocker I, 1 was taking diuretics and 3 were taking angiotensin II. In the T2DM group, 6 were on insulin, 6 were on metformin, 1 on lipid-lowering agents, 2 on ACE inhibitors, 3 on beta-blocker I, 1 on diuretics and 3 on angiotensin II. In Iglesias Molli et al. (30), participants underwent changes in lifestyle to achieve the target metabolic control (HbA1c under 7%). Each participant received personalized pharmacological treatment. The pharmacological treatment of choice in 19 participants was metformin in doses between 500 and 2550 mg/day; 5 participants received combined treatment with metformin and insulin; 1 participant received metformin and glibenclamide; 1 participant received vildagliptin; and 1 participant did not receive pharmacological treatment.

### Genetic analysis

Three studies undertook genetic analysis. Margaryan et al. (15) questioned whether DNA methylation status of *IL1RN*, *RELA (p65)* and *NFKB1 (p50)* genes may be relevant to IL-1β and IL-1Ra production and may be a possible contributory factor in the inflammatory pathogenesis of T2DM (1). Patel et al. (21) performed genotyping of neuropeptide Y (NPY) and IL-1β single nucleotide polymorphisms (SNPs). The allele and genotype frequencies for *IL1B* -511C/T polymorphism were calculated in T2DM and controls. Iglesias Molli et al. (30) evaluated the association between the rs16944 genotype with mRNA expression, serum levels of IL-1β, biochemical and clinical variables.

### Indicators of glucose tolerance and insulin sensitivity

Glucose tolerance and insulin sensitivity were assessed in most studies by different methods. Ten studies measured FPG (15, 16, 21, 22, 24, 26–30), one study (30) only had T2DM group. Eleven studies measured HbA1c (15, 16, 22–30). Four studies (16, 22, 25, 30) only measured HbA1c in the T2DM group. Banerjee et al. (24) measured fasting plasma insulin and Zhou et al. (26) measured fasting serum insulin. Three studies (16, 24, 26) assessed homeostatic model assessment for insulin resistance (HOMA-IR), Tong et al. (16) calculated homeostatic model assessment of β-cell function (HOMA- β) and quantitative insulin-sensitivity check index (QUICKI). Zhou et al. (26) calculated acute insulin response (AIR) and first‐phase insulin secretion (AUC) while (23) conducted a standard meal test (500 kilocalories of energy intake).

### Baseline characteristics

Twelve studies reported the age of participants included (15, 16, 20, 21, 23–30). Margaryan et al. (22) only mentioned the age of the T2DM group and not the HC group and Iglesias Molli et al. (30) only had T2DM participants. Five studies reported a significant difference in the age between the T2DM and healthy controls (HC) (16, 20, 24, 25, 28). Banerjee et al. (24) reported a significant difference in the G1 subgroup (age 31- 40 years). Three studies did not report a significant difference in the age between the T2DM group and HC (23, 26, 27) and Banerjee et al. (24) did not report a significant difference in the G2 subgroup (age 41- 50 years). Four studies did not mention the difference in age between the T2DM group and the HC group. (15, 21, 22, 29)

Twelve studies reported the sex of the participants (15, 16, 20, 21, 23–30). Margaryan et al. (22) only mentioned the sex of the T2DM group and not the HC group and Iglesias Molli et al. (30) only had T2DM participants. Only two studies (16, 23) reported a significant difference in the sex distribution between the T2DM group and the HC group. Four studies did not mention the difference in sex between the T2DM and the HC group (15, 21, 22, 27). In two studies (20, 24) participants were all females while Lima-Cabello et al. (28) participants were all males.

Eleven studies reported BMI (15, 16, 20, 21, 23–26, 28–30). Margaryan et al. (22) only mentioned the BMI of the T2DM group and Iglesias Molli et al. (30) only had T2DM participants. Four studies (21, 23, 26, 28) reported a significant difference in the BMI between the T2DM and the HC group; Banerjee et al. (24) reported a significant difference in the G1 subgroup (age 31- 40 years). Four studies (16, 20, 25, 29) did not report a significant difference in the BMI between the T2DM and the HC group. Three studies (15, 22, 27) did not mention the difference in BMI between the T2DM and the HC groups.

### Baseline lipid profile

Nine studies reported HDL levels (15, 16, 20, 21, 23, 24, 26, 29, 30), Margaryan et al. (22) only mentioned HDL levels in the T2DM group. Two studies (21, 29) reported a significant difference in the HDL levels between groups, while four studies (20, 23, 26, 29) did not report a significant difference in the HDL levels between the T2DM and the HC groups. Two studies (15, 22) did not mention the difference in HDL levels between the T2DM and the HC group.

Nine studies reported the LDL levels (15, 16, 20, 21, 23, 24, 26, 29, 30). Three studies reported a significant difference in the LDL levels between the T2DM and the HC group (16, 21, 23). Four studies did not report a significant difference in LDL levels between the T2DM and the HC group (20, 24, 26, 29).

Nine studies reported triglyceride (TG) and total cholesterol (TC) levels (16, 20, 21, 23–26, 29, 30). Seven studies reported a significant difference in the TG levels between the T2DM and the HC group (16, 21, 23–26, 29). (20) did not report a significant difference in the TG levels between the T2DM group and the HC group. Two studies (16, 23) reported a significant difference in the TC levels between groups. Six studies did not report a significant difference in the TC levels between the T2DM and the HC group (20, 21, 24–26, 29).

## Discussion

**Figure 5:**
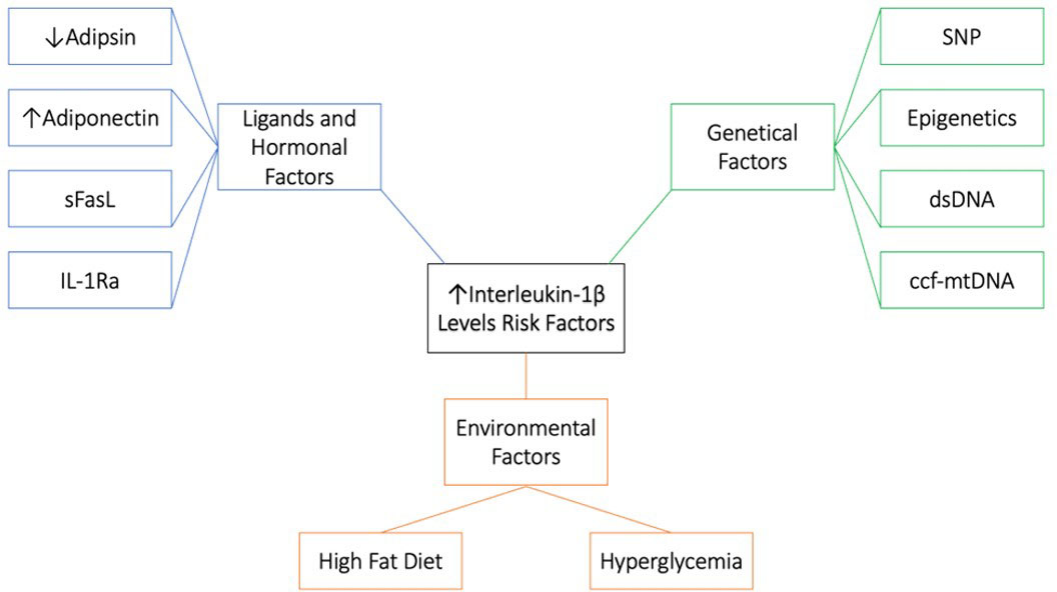
Risk factors associated with high IL-1β levels. 1. Genetical factors; single-nucleotide polymorphism (SNP), Epigenetics, double stranded DNA (dsDNA) and circulating cell-free mitochondrial DNA (ccf-mtDNA). 2. Environmental factors; high fat diet and hyperglycemia. 3. Ligand and hormonal factors; low adipsin levels, high adiponectin levels, soluble Fas ligand (sFasL) and IL-1 receptor antagonist (IL-1Ra).

The current systematic review and meta-analysis covers for the first time a small number of studies assessing IL-1β levels in a T2DM group and in a HC group. Only 13 studies met the pre-defined inclusion criteria, and one of these studies only measured IL-1β in the T2DM group (30). According to these limited studies, IL-1β levels were mostly elevated in the T2DM group and directly proportional with FPG and HbA1c levels. Meta-analysis indicates high heterogeneity between the studies this could be due to the large age scale included in the meta-analysis, differences in the duration of the disease between the patients or differences in the study assay or design. IL-1β levels were also affected by age, BMI and many genetical, hormonal and environmental factors (**Figure 5**). Treatment with either or both metformin and acarbose had a nullifying result on IL-1β levels in the T2DM group.

**Figure 6:**
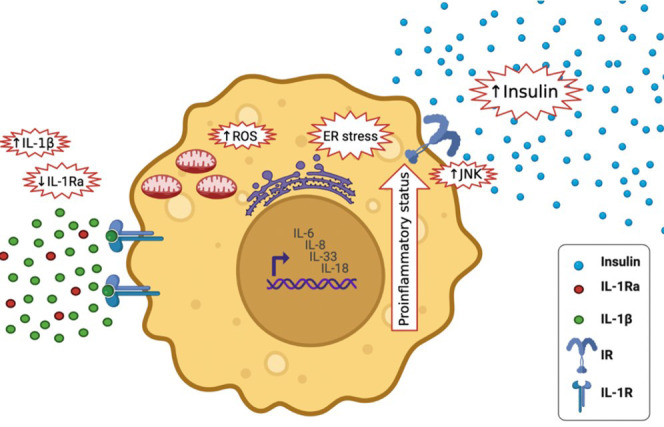
Effects of high IL-1β in T2DM and lower levels of IL-1Ra; it activates the expression of other pro-inflammatory cytokines, including IL-6, IL-8, IL-33 and IL-18, It increases insulin secretion, it stimulates endoplasmic reticulum stress as well as oxidative stress (ROS generation), it stimulates c-Jun N-terminal kinases (JNK).

How can the physiological role of IL-1β switch to a pathological role in β cell failure and apoptosis? There are a couple of explanations for the double-edged sword effect of IL-1β; 1) Lower-levels of IL-1β prevents β-cell apoptosis and stimulates β-cell propagation and glucose activated insulin production. Whereas higher-levels of IL-1β exhaust β cells and have contrary consequences. Thus, IL-1β might play a part in both β-cell apoptosis and propagation (31, 32). IL-1β signaling in β cells is also more prolonged and aperiodic. While momentary exposure to macrophage derived IL-1β can increase insulin levels, prolonged exposure to IL-1β can cause β cell compensation. The signaling shift from the physiological protein kinase C (PKC) and diacylglycerol (DAG) pathways to the pathological mitogen-activated protein kinase (MAPK) and nuclear factor kappa light chain enhancer of activated β cells (NF-κβ) pathways might influence the concentration and time dependent effects of IL-1β exposure (33). 2) Long-lasting activation of β cells by IL-1β may lead to resistance or unresponsiveness. Opposing to β-cells from healthy subjects, those from T2DM patients lack insulin secretion following stimulation by miniscule levels of IL-1β (31). 3) There may be inadequate IL-1Ra in β cells (**Figure 6**). IL-1Ra is a protecting protein stimulated by IL-1β itself to trigger a negative feedback loop (31). 4) Long-lasting activation of β cells by IL-1β may ultimately change the transcriptional program, disturbing the cell’s identity, proliferation, and death (31).

Elevated IL-1β levels in T2DM activates the expression of other inflammatory cytokines, including IL-6, IL-8 (33), IL-33 and IL-18, and amplifying the pro-inflammatory environment (34). It increases insulin secretion temporary, which maybe maleficent to metabolism (35). Furthermore, it stimulates endoplasmic reticulum and oxidative stress, both of which are tightly associated to T2DM (36). It also stimulates c-Jun N-terminal kinases (JNK), activating serine-phosphorylation of insulin receptor substrate-1 (IRS-1) and reducing the activity of insulin-PI3K/PKB signaling pathway in insulin responsive cells (37) (**Figure 6**). During glucose activation, β-cells release IL-1β, IL-1β in a positive feed-back vicious cycle activates its own synthesis in β cells and attracts macrophages act as an additional source of IL-1β and other cytokines (32). Prolonged innate immune stimulation activates diabetes and linked diseases (33).

Obesity is linked to low-grade systemic inflammation that progresses to metabolic diseases as well as insulin resistance accompanied by altered resident immune cells in adipose tissue (38). Obese patients have an upregulation in NLRP3 and IL-1β expression in intra-abdominal and sub-cutaneous fat and this was confirmed by genetic analysis (39–41), eventually leading to NLRP3 inflammasome activation and caspase-1mediated IL-1β and IL-18 secretion and pyroptosis (38). Pyroptosis is an inflammatory type of programmed cell death that is crucial for IL‐1β release because it lacks a signal sequence for exocytosis (42).

Consumption of saturated fatty acids (SFA) is tightly related to obesity and has been correlated with IR and inflammatory diseases in humans. SFA suppresses AMP-activated protein kinase (AMPK) which increases mtROS production and hinders autophagy, leading the activation of the NLRP3 inflammasome and IL-1β driven IR (38). Managing weight and calory intake can contradict this upregulation. Hence, implying that obesity induced insulin resistance (IR) and NLRP3 inflammasome activation are interconnected (39–41). Vandanmagsar et al. outlined an important function of the NLRP3 inflammasome in this inflammatory obesity induced disease where danger associated molecular patterns (DAMP), such as oxidized LDL and cholesterol crystals induce a NLRP3 and IL-1β driven pro-inflammatory reaction (43). Lipopolysaccharides (LPS) are one of the most vigorous pathogen associated molecular patterns “PAMP” for priming the NLRP3 inflammasome, they upregulate the expression of NLRP3 and pro-IL-1β. Blood levels of LPS rise because of increased gut permeability and changes in gut microbiota in T2DM patients who are obese (44).

The authors acknowledge some limitations which should be taken into consideration in the interpretation of the review presented. Only 13 studies met the inclusion criteria and some of these were limited by small sample sizes, one without a HC group, one with all male subjects and one with all female subjects and one study was limited to a certain population. Furthermore, expanding the search up to 2010 led to no additional eligible studies. The mean and standard deviation of the T2DM group and the HC group was only mentioned in three studies. Other pertinent information such as duration of diabetes and physical activity were missing in the majority of studies, which, aside from the different assays used and the characteristics of the population, could have greatly contributed in the high heterogeneity of the analysis.

## Conclusion

In conclusion, the limited studies included in the current systematic review and meta-analysis indicate high but non-significant IL-1β levels in T2DM patients. Variations in assays, sample sizes, population characteristics and design make it difficult to harmonize available data. More studies, both observational and longitudinal, using standardized assays, are necessary to conclude if IL-1β can be a promising biomarker for T2DM.

## Data availability statement

The original contributions presented in the study are included in the article/**supplementary material**. Further inquiries can be directed to the corresponding author.

## Author contributions

HA, SS and NA-D contributed in study conception and design; HA wrote the manuscript; SS, reviewed the manuscript; SS and NA-D supervised the review. All authors contributed to the article and approved the submitted version.

## Funding

The authors are grateful to the Deanship of Scientific Research, King Saud University for funding this research project through Vice Deanship of Scientific Research Chairs.

## Conflict of interest

The authors declare that the research was conducted in the absence of any commercial or financial relationships that could be construed as a potential conflict of interest.

## Publisher’s note

All claims expressed in this article are solely those of the authors and do not necessarily represent those of their affiliated organizations, or those of the publisher, the editors and the reviewers. Any product that may be evaluated in this article, or claim that may be made by its manufacturer, is not guaranteed or endorsed by the publisher.

## References

[1] Cho NH Shaw JE Karuranga S Huang Y Da Rocha Fernandes JD Ohlrogge AW . IDF diabetes atlas: Global estimates of diabetes prevalence for 2017 and projections for 2045. Diabetes Res Clin Pract (2018) 138:271–81. doi: 10.1016/j.diabres.2018.02.023 29496507

[2] Einarson TR Acs A Ludwig C Panton UH . Prevalence of cardiovascular disease in type 2 diabetes: a systematic literature review of scientific evidence from across the world in 2007-2017. Cardiovasc Diabetol (2018) 17:83. doi: 10.1186/s12933-018-0728-6 29884191 PMC5994068

[3] Wang N Zhu F Chen L Chen K . Proteomics, metabolomics and metagenomics for type 2 diabetes and its complications. Life Sci (2018) 212:194–202. doi: 10.1016/j.lfs.2018.09.035 30243649

[4] Sepehri Z Kiani Z Afshari M Kohan F Dalvand A Ghavami S . Inflammasomes and type 2 diabetes: An updated systematic review. Immunol Lett (2017) 192:97–103. doi: 10.1016/j.imlet.2017.10.010 29079203

[5] Yang K Xu S Zhao H Liu L Lv X Hu F . Hypoxia and porphyromonas gingivalis-lipopolysaccharide synergistically induce NLRP3 inflammasome activation in human gingival fibroblasts. Int Immunopharmacol. (2021) 94:107456. doi: 10.1016/j.intimp.2021.107456 33588175

[6] Li G Xia M Abais JM Boini K Li PL Ritter JK . Protective action of anandamide and its COX-2 metabolite against l-Homocysteine-Induced NLRP3 inflammasome activation and injury in podocytes. J Pharmacol Exp Ther (2016) 358:61–70. doi: 10.1124/jpet.116.233239 27189966 PMC4931881

[7] Unamuno X Gomez-Ambrosi J Ramirez B Rodriguez A Becerril S Valenti V . NLRP3 inflammasome blockade reduces adipose tissue inflammation and extracellular matrix remodeling. Cell Mol Immunol (2021) 18:1045–57. doi: 10.1038/s41423-019-0296-z PMC8115140 31551515

[8] Hung YL Wang SC Suzuki K Fang SH Chen CS Cheng WC . Bavachin attenuates LPS-induced inflammatory response and inhibits the activation of NLRP3 inflammasome in macrophages. Phytomedicine (2019) 59:152785. doi: 10.1016/j.phymed.2018.12.008 31009850

[9] Kim SM Kim YG Kim DJ Park SH Jeong KH Lee YH . Inflammasome-independent role of NLRP3 mediates mitochondrial regulation in renal injury. Front Immunol (2018) 9:2563. doi: 10.3389/fimmu.2018.02563 30483252 PMC6240646

[10] Lebreton F Berishvili E Parnaud G Rouget C Bosco D Berney T . NLRP3 inflammasome is expressed and regulated in human islets. Cell Death Dis (2018) 9:726. doi: 10.1038/s41419-018-0764-x 29941940 PMC6018156

[11] Ward R Li W Abdul Y Jackson L Dong G Jamil S . NLRP3 inflammasome inhibition with MCC950 improves diabetes-mediated cognitive impairment and vasoneuronal remodeling after ischemia. Pharmacol Res (2019) 142:237–50. doi: 10.1016/j.phrs.2019.01.035 PMC6486792 30818045

[12] Sokolova M Sahraoui A Hoyem M Ogaard J Lien E Aukrust P . NLRP3 inflammasome mediates oxidative stress-induced pancreatic islet dysfunction. Am J Physiol Endocrinol Metab (2018) 315:E912–23. doi: 10.1152/ajpendo.00461.2017 30016155

[13] King BC Kulak K Krus U Rosberg R Golec E Wozniak K . Complement component C3 is highly expressed in human pancreatic islets and prevents beta cell death *via* ATG16L1 interaction and autophagy regulation. Cell Metab (2019) 29:202–210 e6. doi: 10.1016/j.cmet.2018.09.009 30293775

[14] Herder C Dalmas E Boni-Schnetzler M Donath MY . The IL-1 pathway in type 2 diabetes and cardiovascular complications. Trends Endocrinol Metab (2015) 26:551–63. doi: 10.1016/j.tem.2015.08.001 26412156

[15] Margaryan S Kriegova E Fillerova R Smotkova Kraiczova V Manukyan G . Hypomethylation of IL1RN and NFKB1 genes is linked to the dysbalance in IL1beta/IL-1Ra axis in female patients with type 2 diabetes mellitus. PloS One (2020) 15:e0233737. doi: 10.1371/journal.pone.0233737 32470060 PMC7259508

[16] Tong HV Luu NK Son HA Hoan NV Hung TT Velavan TP . Adiponectin and pro-inflammatory cytokines are modulated in Vietnamese patients with type 2 diabetes mellitus. J Diabetes Investig (2017) 8:295–305. doi: 10.1111/jdi.12579 PMC5415486 27684566

[17] Wang Y Che M Xin J Zheng Z Li J Zhang S . The role of IL-1beta and TNF-alpha in intervertebral disc degeneration. BioMed Pharmacother (2020) 131:110660. doi: 10.1016/j.biopha.2020.110660 32853910

[18] Kammoun HL Allen TL Henstridge DC Barre S Coll RC Lancaster GI . Evidence against a role for NLRP3-driven islet inflammation in db/db mice. Mol Metab (2018) 10:66–73. doi: 10.1016/j.molmet.2018.02.001 29478918 PMC5985230

[19] Liberati A Altman DG Tetzlaff J Mulrow C Gotzsche PC Ioannidis JP . The PRISMA statement for reporting systematic reviews and meta-analyses of studies that evaluate health care interventions: explanation and elaboration. J Clin Epidemiol (2009) 62:e1–34. doi: 10.1016/j.jclinepi.2009.06.006 19631507

[20] Borges L Passos MEP Silva MBB Santos VC Momesso CM Pithon-Curi TC . Dance training improves cytokine secretion and viability of neutrophils in diabetic patients. Mediators Inflammation (2019) 2019:2924818. doi: 10.1155/2019/2924818 PMC6886327 31827375

[21] Patel R Dwivedi M Mansuri MS Ansarullah Laddha NC Thakker A . Association of neuropeptide-y (NPY) and interleukin-1beta (IL1B), genotype-phenotype correlation and plasma lipids with type-II diabetes. PloS One (2016) 11:e0164437. doi: 10.1371/journal.pone.0164437 27749914 PMC5066977

[22] Margaryan S Witkowicz A Arakelyan A Partyka A Karabon L Manukyan G . sFasL-mediated induction of neutrophil activation in patients with type 2 diabetes mellitus. PloS One (2018) 13:e0201087. doi: 10.1371/journal.pone.0201087 30024959 PMC6053218

[23] Mo D Liu S Ma H Tian H Yu H Zhang X . Effects of acarbose and metformin on the inflammatory state in newly diagnosed type 2 diabetes patients: a one-year randomized clinical study. Drug Des Devel. Ther (2019) 13:2769–76. doi: 10.2147/DDDT.S208327 PMC6691948 31496653

[24] Banerjee J Dhas Y Mishra N . Middle-aged indians with type 2 diabetes are at higher risk of biological ageing with special reference to serum CDKN2A. J Diabetes Res (2020) 2020:7569259. doi: 10.1155/2020/7569259 32280716 PMC7128035

[25] Bae JH Jo SI Kim SJ Lee JM Jeong JH Kang JS . Circulating cell-free mtDNA contributes to AIM2 inflammasome-mediated chronic inflammation in patients with type 2 diabetes. Cells (2019) 8. doi: 10.3390/cells8040328 PMC6524162 30965677

[26] Zhou Q Ge Q Ding Y Qu H Wei H Wu R . Relationship between serum adipsin and the first phase of glucose-stimulated insulin secretion in individuals with different glucose tolerance. J Diabetes Investig (2018) 9:1128–34. doi: 10.1111/jdi.12819 PMC6123022 29432659

[27] Piarulli F Sartore G Sechi A Basso D Fogar P Greco E . Low glucose concentrations induce a similar inflammatory response in monocytes from type 2 diabetic patients and healthy subjects. Oxid Med Cell Longev. (2017) 2017:9185272. doi: 10.1155/2017/9185272 29225725 PMC5684594

[28] Lima-Cabello E Alche V Foley RC Andrikopoulos S Morahan G Singh KB . Narrow-leafed lupin (Lupinus angustifolius l.) beta-conglutin proteins modulate the insulin signaling pathway as potential type 2 diabetes treatment and inflammatory-related disease amelioration. Mol Nutr Food Res (2017) 61. doi: 10.1002/mnfr.201600819. 28012244

[29] Torres M Herrera MT Fabian-San-Miguel G Gonzalez Y . The intracellular growth of m. tuberculosis is more associated with high glucose levels than with impaired responses of monocytes from T2D patients. J Immunol Res (2019) 2019:1462098. doi: 10.1155/2019/1462098 31815150 PMC6877949

[30] Iglesias Molli AE Bergonzi MF Spalvieri MP Linari MA Frechtel GD Cerrone GE . Relationship between the IL-1beta serum concentration, mRNA levels and rs16944 genotype in the hyperglycemic normalization of T2D patients. Sci Rep (2020) 10:9985. doi: 10.1038/s41598-020-66751-x 32561825 PMC7305205

[31] Boni-Schnetzler M Meier DT . Islet inflammation in type 2 diabetes. Semin Immunopathol (2019) 41:501–13. doi: 10.1007/s00281-019-00745-4 PMC6592966 30989320

[32] Zhao G Dharmadhikari G Maedler K Meyer-Hermann M . Possible role of interleukin-1beta in type 2 diabetes onset and implications for anti-inflammatory therapy strategies. PloS Comput Biol (2014) 10:e1003798. doi: 10.1111/jdi.12819 25167060 PMC4148195

[33] Donath MY Dinarello CA Mandrup-Poulsen T . Targeting innate immune mediators in type 1 and type 2 diabetes. Nat Rev Immunol (2019) 19:734–46. doi: 10.1038/s41577-019-0213-9 31501536

[34] Arend WP Palmer G Gabay C . IL-1, IL-18, and IL-33 families of cytokines. Immunol Rev (2008) 223:20–38. doi: 10.1111/j.1600-065X.2008.00624.x 18613828

[35] Dror E Dalmas E Meier DT Wueest S Thevenet J Thienel C . Postprandial macrophage-derived IL-1beta stimulates insulin, and both synergistically promote glucose disposal and inflammation. Nat Immunol (2017) 18:283–92. doi: 10.1038/ni.3659 28092375

[36] Verma G Datta M . IL-1beta induces ER stress in a JNK dependent manner that determines cell death in human pancreatic epithelial MIA PaCa-2 cells. Apoptosis (2010) 15:864–76. doi: 10.1007/s10495-010-0498-4 20411335

[37] Banerjee M Saxena M . Interleukin-1 (IL-1) family of cytokines: role in type 2 diabetes. Clin Chim Acta (2012) 413:1163–70. doi: 10.1016/j.cca.2012.03.021 22521751

[38] Wani K Alharthi H Alghamdi A Sabico S Al-Daghri NM . Role of NLRP3 inflammasome activation in obesity-mediated metabolic disorders. Int J Environ Res Public Health (2021) 18:511. doi: 10.3390/ijerph18020511 PMC7826517 33435142

[39] Moschen AR Molnar C Enrich B Geiger S Ebenbichler CF Tilg H . Adipose and liver expression of interleukin (IL)-1 family members in morbid obesity and effects of weight loss. Mol Med (2011) 17:840–5. doi: 10.2119/molmed.2010.00108 PMC3146615 21394384

[40] Mocanu AO Mulya A Huang H Dan O Shimizu H Batayyah E . Effect of roux-en-Y gastric bypass on the NLRP3 inflammasome in adipose tissue from obese rats. PloS One (2015) 10:e0139764. doi: 10.1371/journal.pone.0139764 26437377 PMC4593548

[41] Al-Daghri n. M. Wani K Alharthi H Alghamdi A Alnaami AM Yakout SM . Sex-specific signature in the circulating NLRP3 levels of Saudi adults with metabolic syndrome. J Clin Med (2021) 10:3288. doi: 10.3390/jcm10153288 34362072 PMC8347773

[42] Watanabe S Usui-Kawanishi F Karasawa T Kimura H Kamata R Komada T . Glucose regulates hypoxia-induced NLRP3 inflammasome activation in macrophages. J Cell Physiol (2020) 235:7554–66. doi: 10.1002/jcp.29659 32115713

[43] Vandanmagsar B Youm YH Ravussin A Galgani JE Stadler K Mynatt RL . The NLRP3 inflammasome instigates obesity-induced inflammation and insulin resistance. Nat Med (2011) 17:179–88. doi: 10.1038/nm.2279 PMC3076025 21217695

[44] Cani PD Amar J Iglesias MA Poggi M Knauf C Bastelica D . Metabolic endotoxemia initiates obesity and insulin resistance. Diabetes (2007) 56:1761–72. doi: 10.2337/db06-1491 17456850

