# Supplementary material for: The role of interleukin-1β in type 2 diabetes mellitus: A systematic review and meta-analysis
Source: Front Endocrinol (Lausanne). 2022 Jul 27;13:901616. doi: 10.3389/fendo.2022.901616 (PMC9363617; doi:10.3389/fendo.2022.901616)
Supplement: Supplementary file 1 [file Table_1.docx]

**Supplementary Table 1.** Summary of Critical Appraisal

| - **Study** | - **Design** | **Q1** | **Q2** | **Q3** | **Q4** | **Q5** | **Q6** | **Q7** | **Q8** | **Q9** | **Q10** | **Q11** | **Q12** |
| --- | --- | --- | --- | --- | --- | --- | --- | --- | --- | --- | --- | --- | --- |
| Margaryan et al. 2020 | - Case Control | ✓ | ✓ | ? | ✓ | ✓ | x | - | - | ✓ | x | ? |  |
| Margaryan et al. 2018 | - Case Control | ✓ | ✓ | ? | x | ✓ | x | - | - | x | x | ✓ |  |
| Tong et al. 2017 | - Case Control | ✓ | ✓ | ? | ? | ✓ | ✓ | - | - | ✓ | x | ✓ |  |
| Mo et al. 2019 | - Randomized Controlled Trial | ✓ | ? | ✓ | ? | x | x | ✓ | x | ✓ | ✓ | ✓ |  |
| Banerjee, et al 2020 | - Case Control | ✓ | ✓ | ✓ | ? | ✓ | x | - | - | x | x | ? |  |
| Patel et al. 2016 | - Case Control | ✓ | ✓ | ✓ | ? | ✓ | ✓ | - | - | ✓ | ? | ? |  |
| Bae et al. 2019 | - Case Control | ✓ | ✓ | ? | x | ? | x | - | - | x | ? | ✓ |  |
| Zhou et al. 2018 | - Case Control | ✓ | ✓ | ✓ | ✓ | ✓ | x | - | - | ✓ | ? | ✓ |  |
| Piarulli et al. 2017 | - Case Control | ✓ | ✓ | ? | ? | ? | ✓ | - | - | x | ? | ? |  |
| Lima-Cabello et al. 2017 | - Case Control | ✓ | ✓ | ? | ? | ? | x | ✓ | ✓ | ✓ | x | ? |  |
| Borges et al. 2019 | - Randomized Controlled Trial | ✓ | x | ✓ | x | x | ✓ | ✓ | x | ✓ | ✓ | ? |  |
| Torres et al. 2019 | - Case Control | ✓ | ✓ | ✓ | x | ✓ | ? | - | - | ✓ | ? | ? |  |
| Iglesias Molli et al. 2020 | - Cohort | ✓ | ✓ | ? | ✓ | ✓ | x | ? | ? | ? | ✓ | ✓ | ? |

‘✓’ (yes); ‘x’ (no); ‘?’ (can’t tell); ‘- ‘(not applicable)

**Case Control Study:**

1. Did the study address a clearly focused issue?
2. Did the authors use an appropriate method to answer their question?
3. Were the cases recruited in an acceptable way?
4. Were the controls selected in an acceptable way?
5. Was the exposure accurately measured to minimise bias?
6. (a) Aside from the experimental intervention, were the groups treated equally?

(b) Have the authors taken account of the potential confounding factors in the design and/or in their analysis?

1. How large was the treatment effect?
2. How precise was the estimate of the treatment effect?
3. Do you believe the results?
4. Can the results be applied to the local population?
5. Do the results of this study fit with other available
   evidence?

**Randomised Controlled Trial:**

1. Did the study address a clearly focused research question?
2. Was the assignment of participants to interventions randomised?
3. Were all participants who entered the study accounted for at its conclusion?
4. •Were the participants ‘blind’ to intervention they were given?

• Were the investigators ‘blind’ to the intervention they were giving to participants?

• Were the people assessing/analysing outcome/s ‘blinded’?

1. Were the study groups similar at the start of the randomised controlled trial?
2. Apart from the experimental intervention, did each study group receive the same level of care (that is, were they treated equally)?
3. Were the effects of. Intervention reported comprehensively?
4. Was the precision of the estimate of the intervention or treatment effect reported?
5. Do the benefits of the experimental intervention outweigh the harms and costs?
6. Can the results be applied to your local population/in your context?
7. Would the experimental intervention provide greater value to the people in your care than any of the existing interventions?

**Cohort Study**

1. Did the study address a clearly focused issue?
2. Was the cohort recruited in an acceptable way?
3. Was the exposure accurately measured to minimise bias?
4. Was the outcome accurately measured to minimise bias?
5. (a) Have the authors identified all important confounding factors?

(b) Have they taken account of the confounding factors in the design and/or analysis?

1. (a) Was the follow up of subjects complete enough?

(b) Was the follow up of subjects long enough?

1. What are the results of this study?
2. How precise are the results?
3. Do you believe the results?
4. Can the results be applied to the local population?
5. Do the results of this study fit with other available evidence?
6. What are the implications of his study for practice?
